# Airflow Dynamics for Micro-Wind Environment Optimization and Human Comfort Improvement: Roadshow Design for Theater Stage Spaces

**DOI:** 10.3390/s25144456

**Published:** 2025-07-17

**Authors:** Yiheng Liu, Menglong Zhang, Wenyang Han, Yufei He, Chang Yi, Yin Zhang, Jin Li

**Affiliations:** 1 School of Architecture, Southwest Minzu University, Chengdu 610225, China; ianno_0201@163.com (Y.L.); z18768972650@163.com (M.Z.); 15882067415@163.com (W.H.); hyf136748235062023@163.com (Y.H.); isyichang@163.com (C.Y.); 2 AI + Arch Laboratory, Southwest Minzu University, Chengdu 610225, China; 3 School of Civil Engineering, Sichuan University of Science & Engineering, Zigong 643000, China

**Keywords:** airflow distribution, indoor ventilation, human comfort, optimization design, computational fluid dynamics, CFD

## Abstract

The optimization of ventilation strategies in high-ceiling theater stage spaces is crucial for improving thermal comfort and energy efficiency. This study addresses the challenge of uneven temperature distribution and airflow stagnation in stage environments by employing computational fluid dynamics (CFD) simulations to evaluate the effectiveness of different ventilation modes, including natural, mechanical, and hybrid systems. Six airflow organization scenarios were designed based on modifications to structural layout, equipment settings, and mechanical disturbances (e.g., fan integration). Key evaluation indicators such as temperature uniformity coefficient, airflow velocity, and exhaust efficiency were used to assess performance. The results show that a multi-dimensional optimization approach combining spatial adjustments and mechanical disturbances significantly reduced the average temperature from 26 °C to 23 °C and the temperature uniformity coefficient from 2.79 to 1.49. This study contributes a comprehensive design strategy for stage ventilation that improves comfort while minimizing energy consumption, offering practical implications for performance space design and HVAC system integration.

## 1. Introduction

### 1.1. Background

With the growing demand for high-performance public cultural facilities, modern theaters have evolved into complex spaces that must balance functionality, occupant comfort, and energy efficiency [1]. Among these, the stage space—a core functional zone—presents unique thermal and ventilation challenges due to high occupant density, dense lighting equipment, and semi-enclosed architectural layouts. These characteristics often lead to thermal stratification, heat accumulation, and airflow stagnation, severely compromising thermal comfort for performers and equipment safety. While previous studies have explored general ventilation strategies and HVAC solutions for large spaces, most research has concentrated on lecture halls or auditoriums, with relatively limited focus on stage-specific spatial conditions [2]. Existing approaches also often address single-variable optimizations [3,4], lacking an integrated methodology that accounts for spatial configuration, equipment layout, and active airflow control [5]. In traditional theater design, the thermal environment of the stage space is often overlooked, leading to issues such as temperature unevenness and poor air circulation. These problems can affect the comfort and performance of the performers and may even impact the overall quality of the production [6]. The stage area typically accommodates a large number of lights, set pieces, and electrical equipment. While these factors contribute to an increased thermal load, they can also lead to uneven temperature distribution [7]. To improve the thermal environment of theater stage areas, researchers have gradually integrated airflow organization with thermal environment optimization. Proper airflow distribution not only reduces thermal load and temperature differences but also provides more efficient ventilation, exhaust, and cooling effects [8]. With the advancement of architectural design concepts and the development of simulation technologies, an increasing number of studies have begun to employ numerical simulations, experimental research, and case analyses to explore various methods and strategies for optimizing airflow organization in stage spaces [9]. However, although existing studies have provided a theoretical foundation for optimizing the thermal environment of theater spaces, research specifically focused on the unique environment of the theater stage remains relatively limited [10]. This gap leaves the question of how to systematically optimize airflow distribution in complex stage environments largely unresolved. Therefore, this study aims to bridge this gap by proposing a multi-dimensional optimization strategy that combines computational fluid dynamics (CFD) simulations, architectural layout analysis, and perturbation techniques to improve airflow performance and thermal comfort in theater stage spaces. The main research objectives are as follows:

(1) To identify the limitations of traditional ventilation and natural ventilation in stage environments.

(2) To develop and simulate multiple airflow improvement scenarios based on structure, equipment, and active disturbance.

(3) To evaluate each scenario using key performance indicators such as temperature uniformity, airflow distribution, and exhaust efficiency.

In this study, computational fluid dynamics (CFD) is employed as a key simulation tool to analyze airflow patterns and temperature distribution within the stage space. CFD allows for the visualization and quantitative evaluation of different ventilation strategies, thereby providing a theoretical foundation for optimizing the thermal environment in large, enclosed public buildings such as theaters.

### 1.2. Literature Review

Currently, research on optimizing the thermal environment of stage spaces is relatively limited, with existing studies primarily focusing on building ventilation, air conditioning design, and thermal comfort. Many studies have explored the impact of ventilation methods and airflow organization on the thermal environment of stage spaces. Nada et al. [10] use a 3D-CFD investigation of airflow, temperature distribution, and thermal comfort in high-rise ceiling theaters air-conditioned with underfloor air distribution (UFAD) systems for different operating and geometric conditions. Cheong et al. [11] evaluates the current thermal comfort conditions of an air-conditioned lecture theater in a tertiary institution using objective measurement, computational fluid dynamics (CFD) modeling, and subjective assessment. The characteristics of turbulence (turbulence intensity, turbulence length scale, turbulent kinetic energy, etc.) were investigated in 20 typical ventilation spaces by Hanzawa et al. [12]. It was found that the mean velocity and turbulence intensity varied greatly across the ventilation spaces, with mean velocities ranging from less than 0.05 m/s to 0.40 m/s and turbulence intensities ranging from 10% to 70%. Mel’kumov et al. [13] established an airflow model for audience replacement ventilation based on the theory of equirectangular mapping, which improves the accuracy of airflow analysis using elliptic integrals and provides a reliable method for optimizing the ventilation design of large spaces. Taking the airflow distribution of a low sidewall air supply system as the research object, Huang Chen et al. [14] predicted the indoor thermal environment distribution based on the Block–Gebhart (B-G) model. The indoor air temperature, interior wall temperature, and stratified air-conditioning load were found to be −5.440% to 2.473%, −5.024% to 3.277%, and −6.47% to 4.04%, respectively.

Additionally, research on airflow organization in theater stage spaces has predominantly focused on ventilation mode analysis, with a lack of multi-factor, multi-level systematic evaluations. Hwang et al. [15] presents an integrated CFD–neural network–genetic algorithm methodology that significantly reduces computational cost while optimizing DAWT design, revealing rotor axial position as the most influential factor in enhancing power output and balancing airflow acceleration with noise control. Liu et al. [16] identifies key architectural strategies—such as optimizing room depth, opening placement, and window ratios—that significantly enhance natural ventilation in elderly care facilities, offering evidence-based design guidance to improve indoor air quality and comfort in the context of aging and post-pandemic resilience. Du et al. [17] proposes a multi-stage optimization method integrating CFD, surrogate modeling, and genetic algorithms to identify optimal lift-up building designs in urban canyons, effectively enhancing pedestrian wind conditions and outdoor thermal comfort for sustainable urban planning. Wu et al. [18] build a 1:38 small-scale model of a large-scale sphere-shaped facility with high heat flux dissipation for investigating and analyzing the airflow distribution in the upper occupied zone. They found that a supply air temperature of 18 °C was selected as the optimal design parameter after conducting experiments to maintain the upper occupied zone temperature at 21 °C ± 1 °C, and the corresponding minimum airflow rate could significantly reduce the energy consumption of air processing. Kavgic et al. [19] analyzed the level of indoor air quality and thermal comfort in a typical medium-sized mechanically ventilated theater, and to identify where improvements could typically be made, a comprehensive post-occupancy evaluation study was carried out on a theater in Belgrade. They found the calculated ventilation rates showed that the theater was over-ventilated, which will have serious consequences for its energy consumption, and secondly, the displacement ventilation arrangement employed led to higher than expected complaints of cold discomfort, probably due to cold drafts around the occupants’ feet. Noh et al. [20] focused on thermal comfort and indoor air quality in a lecture theater with a four-way cassette air conditioning and mixing ventilation system. This showed that increasing the discharge angle from the supply grilles on the cassette unit makes uniformity of thermal comfort worse but rarely affects IAQ. Papakonstantinou et al. [21], solely based on computational fluid dynamics (CFD) modeling scenarios, showed how this methodology could be used to investigate IAQ issues in theaters. It attempted to evaluate how two ventilation systems with the same air inlet arrangement but different systems of air extraction affected the air speed, temperature, and CO_2_ concentration profile inside the teaching auditorium. The conclusion, not surprisingly, was that the lowest rate of air change leads to an increase in temperature. Furthermore, it was found that CO_2_ concentration decreases rapidly if the ventilation rate is increased, in this case by the unexpectedly large factor of five. Tu et al. [22] used the Grand Canal Exhibition Center as the research object in this study. Its numerical simulation of two pre-designed cases using CFD techniques is carried out, and it studies the indoor airflow organization and temperature field distribution. The results of the research show that case 2 has greater thermal comfort uniformity and energy-saving potential, with a ground floor ADPI value of 83.3% and uniform thermal comfort throughout the entire zone. To investigate the internal natural ventilation during the design process of an office, Fu et al. [23] used CFD simulation software design with or without impact, and measured data were used to confirm the simulation’s reliability. Chiu et al. [24] demonstrated the use of CFD modeling as a tool for scientific modeling to evaluate natural ventilation and interior air quality in green structures. Nada et al. [25] confirms that fully enclosed cold aisle containment significantly enhances thermal performance in data centers, with performance improvements becoming more pronounced at higher power densities. Losi et al. [26] demonstrates that with strategic façade design and a buoyancy-assisted air conditioning system, thermal neutrality and player safety can be achieved in semi-open stadiums under extreme Qatari climate conditions, even with significantly reduced cooling loads. Cho et al. [27] find that vertical aisle partition systems, when evaluated using performance metrics such as Rack Cooling Index (RCI) and Return Temperature Index (RTI), can significantly improve cooling efficiency and reliability in high-density data centers by minimizing air mixing and enabling standardized performance assessment. Alajmi et al. [28] confirms that UFAD systems can reduce energy consumption by approximately 30% compared to CBAD in hot climates, particularly in high-ceiling buildings, while maintaining acceptable thermal comfort through effective use of thermal stratification. Although existing research methods provide valuable insights, issues such as incomplete consideration of factors and insufficient computational accuracy still exist. Therefore, how to comprehensively optimize airflow organization across multiple dimensions, including equipment, structure, and disturbances, remains a critical research challenge that needs to be addressed.

### 1.3. Research Focus

The performance area of a theater stage often experiences thermal non-uniformity, air stagnation, and residual heat buildup due to high internal loads from lighting and equipment, along with architectural features such as tall ceilings and partially enclosed structures. These conditions make it difficult for traditional HVAC systems to maintain comfort without excessive energy use. Moreover, existing studies tend to treat ventilation design in large spaces generically, without addressing the specific airflow and thermal behavior of stage environments.

To address this gap, this study investigates how to improve airflow organization by systematically integrating spatial structural changes, equipment adjustment, and mechanical disturbance techniques. Using CFD simulations, six representative scenarios are evaluated through quantitative indices (temperature uniformity, airflow vectors, and exhaust efficiency) to assess performance improvements. The results offer design guidance for optimizing indoor airflow in complex performance spaces, providing a reference for practical HVAC integration in cultural buildings. The key areas of focus include the following: 1. An analysis of the relationship between airflow distribution characteristics and thermal load in the stage space. 2. A comprehensive consideration of the thermal comfort requirements for performers at a height of 1.5 m. 3. The thermal environment effects of optimization strategies through numerical simulations. 4. An evaluation of the impact of equipment and structure optimization on stage temperature. The innovation of this study lies in the proposal of an airflow optimization solution that comprehensively considers equipment, structure, and disturbances. By combining numerical simulations with real-case scenarios, this study provides a new approach and method for optimizing the thermal environment of theater stage spaces.

## 2. Materials and Methods

### 2.1. Design Code

The design and construction of large public buildings must strictly adhere to national and local building codes, fire safety regulations, energy-saving standards, and environmental protection requirements. In China, common building codes include the “Unified Standard for Building Drawings” (GB/T 50001-2017) [29], “Design standard for energy efficiency of public buildings” (GB 50189-2015) [30], and “Code for fire protection design of buildings ” (GB 50016-2014) [31]. The formulation of each code aims to ensure structural safety, fire safety, energy efficiency, and a comfortable environment in buildings. This is especially crucial in large public buildings, where compliance with these standards is paramount. For instance, in terms of functional zoning, the Uniform standard for design of civil buildings ” (GB 50352-2012) [32] specifies that the airflow velocity in the performance area should be between 0.6 and 1.2 m/s, while in the audience area, it should range from 0.15 to 0.3 m/s [33]. In large public buildings, a well-designed ventilation system is key to ensuring air quality and thermal comfort. According to the “Design code for heating ventilation and air conditioning of industrial buildings” (GB 50019-2015) [33] and related environmental standards, the ventilation system of a building must meet the following requirements: different functional areas should adopt different ventilation methods, such as natural ventilation, mechanical ventilation, or mixed ventilation, to ensure smooth air circulation and meet the required air quality standards [33]. Based on the building’s usage function and occupant density, the ventilation system design should provide sufficient air exchange rates. Modern architectural design increasingly emphasizes energy efficiency and sustainability. The design and construction of large public buildings should comply with the “Assessment standard for green building ” (GB/T 50378-2019) [34] and the “Building Energy Efficiency Design Standard”. During the building design and operation process, efforts should be made to achieve green building rating standards to enhance the building’s sustainability. The illumination standards for different functional areas must adhere to the provisions of the “Lighting of indoor work places”. To improve lighting energy efficiency and comfort, it is recommended that large public buildings be equipped with automated control systems, such as light-sensing adjustment systems and motion sensor switches, to automatically adjust indoor lighting intensity in response to changes in natural light [35]. At the same time, to avoid shadows and uneven brightness, lighting design should ensure uniform illumination within indoor spaces. Generally, the uniformity coefficient of lighting (i.e., the ratio of maximum to minimum illuminance) should be lower than 3:1 to avoid overly concentrated or dimly lit areas. However, traditional standards may overly rely on static environmental parameters, such as steady-state values of temperature, humidity, wind speed, and radiant temperature, while neglecting the impact of dynamic changes and non-uniform environments. For instance, stage lighting can create localized high temperatures, while the audience area remains cooler. Existing standards may not fully account for such spatial unevenness.

### 2.2. Ventialation Principle

Good ventilation design is crucial for air quality, thermal comfort, and the health of occupants in large public buildings. In this study, rather than applying general empirical formulas, ventilation design is addressed through CFD-based simulations, where airflow rates and temperature effects are analyzed numerically under realistic boundary and heat load conditions. Good ventilation design not only focuses on air quality but also involves the regulation of temperature, humidity, and airflow, directly influencing thermal comfort and the health of the indoor environment. By applying thermal comfort calculation formulas (such as the PMV index), the airflow and thermal environment within a building can be scientifically assessed and optimized. Combining accurate calculations of occupant requirements and thermal environment creation techniques helps improve the comfort of living or working spaces while enhancing the efficiency of building energy usage.

### 2.3. CFD Modeling

In this study, computational fluid dynamics (CFD) simulation methods were used to model the airflow distribution and thermal environment changes in the theater stage space. Through numerical simulations, we were able to accurately predict the thermal environment response of the stage space under different airflow strategies, providing a theoretical basis for practical design. Airpak 3.0 software was used for three-dimensional fluid dynamics analysis during the simulation, modeling the impact of various airflow modes on the thermal environment of the stage.

#### 2.3.1. Governing Equations

The numerical simulation in this study is based on fundamental governing equations for fluid flow and heat transfer, including continuity, momentum, energy, and turbulence transport equations. These equations, as applied in Airpak, are summarized in Appendix A. When the flow is laminar, Airpak describes air movement by solving the continuity equation, momentum equation, species transport equation, and energy conservation equation. When the flow is turbulent or includes radiative heat transfer, additional transport equations must be solved [36].

The CFD simulations in this study were performed using Airpak 3.0, a widely used simulation platform based on the Fluent solver and equipped with built-in turbulence and radiation models tailored for indoor airflow analysis. Although experimental validation was beyond the scope of this study, the accuracy of Airpak in modeling large indoor spaces has been demonstrated in previous works.

For example, Li et al. [37] used the Airpak software to establish physical and numerical models for the research object and conducted numerical studies on the indoor environment under different ventilation conditions. It has been found that the displacement ventilation method is significantly superior to the traditional up-and-down ventilation in improving indoor air quality. Feng et al. [38] used the Airpak software to conduct numerical simulation analysis on the air volume distribution and thermal comfort of the side supply air mode of the winter fan coil units. The comparison between the simulation results and the actual measurement results showed a consistent trend, verifying the feasibility and accuracy of Airpak for analyzing the indoor thermal environment comfort. These studies provide confidence in the tool’s application for simulating stratified airflow and thermal comfort in enclosed high-volume environments similar to the stage space considered here.

#### 2.3.2. Boundary Conditions and Parameter Setting

**Figure 1:**
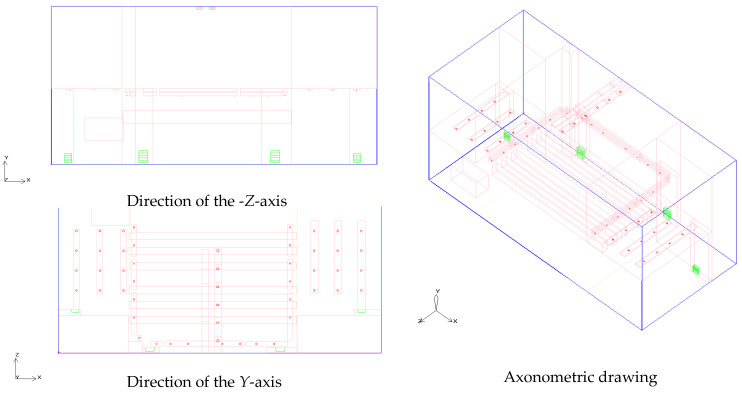
Schematic diagram of the fresh air system in the stage performance area.

The fresh air system of the project consists of four vents located at the rear of the stage space, three rows of exhaust vents in the stage waiting area, two rows of exhaust vents in the performance area, and a mechanical exhaust vent above the central stage space, as shown in Figure 1. This study simulates the airflow and thermal environment response of the theater stage space by carefully setting parameters such as the air supply velocity, boundary conditions for the calculation domain, and the balance conditions for thermal and airflow equilibrium [39].

The setting of the air supply velocity has a direct impact on airflow distribution and the uniformity of the thermal environment. The air velocity determines the driving force of the airflow and the distribution pattern of air within the stage. In this study, the air supply velocity is set to different values to simulate the effects under various ventilation strategies. By adjusting the air supply velocity and flow rate, the airflow within the stage area is ensured to meet the required standards. Based on the airflow and the needs of the stage space, the air supply volume is set within a specific range. In Table 1.

The air supply vents are named along the positive *x*-axis direction from left to right and from top to bottom as opening.1-1 to opening.13-6. The exhaust vent temperature is uniformly set to 20 °C. The air supply velocity is calculated based on the indoor and outdoor wind balance, and then the exhaust velocity for each group and each exhaust vent is determined. The wind balance formula is as follows:

(1) $$Q_{supply}=Q_{retrun}+Q_{exhaust}\pm Q_{infiltration}$$

$Q_{supply}$: total air supply volume, m^3^/h.

$Q_{retrun}$: mechanical return air volume, m^3^/h.

$Q_{exhaust}$: active exhaust air volume, m^3^/h.

$Q_{infiltration}$: passive infiltration air volume through door and window gaps, m^3^/h; it can be estimated using the air change rate method. The air supply volume should be equal to the algebraic sum of the return air volume, exhaust air volume, and infiltration air volume (positive for infiltration and negative for exfiltration).

It should be noted that this study adopts a steady-state simulation approach, in which temperature variation is not applied dynamically over time. Instead, temperature differences emerge from constant internal heat fluxes associated with equipment and lighting and from the air supply at fixed inlet temperatures. The thermal response of the space is calculated under static boundary conditions, with no temporal temperature ramping or transient simulation involved. This method allows for comparative evaluation across scenarios under standardized operating assumptions. The boundary conditions for the calculation domain mainly include the geometric shape of the simulation area and the interface between the stage space and the external environment. The external boundaries of the calculation domain (such as the stage windows, glass curtain walls, etc.) are set as atmospheric boundary conditions. The boundary heat flux is calculated based on external temperature, wind speed, and solar radiation intensity, which influence the air temperature and flow state within the stage. Key boundary conditions are set as shown in Table 2.

The external heat flux values listed in Table 2 were estimated based on standard thermal performance assumptions for building envelope surfaces, following the guidance provided in the Chinese national standard GB 50176-2016 (*Thermal Design Code for Civil Buildings*) [40]. These values take into account the surface orientation, material type, assumed external environmental temperature, and solar radiation intensity for Chengdu’s typical summer conditions. For example, the 110 W/m^2^ value assigned to the mid-floor accounts for cumulative internal and external gains due to direct solar load and equipment load below. Surfaces with lower exposure or shading were assigned conservative values (e.g., 40 W/m^2^). For simulation feasibility, these values were treated as fixed steady-state boundary inputs rather than dynamic loads.

Heat within the room can be transferred through radiative heat transfer and convective heat transfer between the outer surface of the external walls and the surrounding environment. The inner surface of the external walls can also transfer heat to objects within the room through convection or radiation. The convective heat transfer boundary condition can be written as follows:

(2) $$q_{conv}=h_{c}(T_{surface}-T_{air})$$

$q_{conv}$ is the convective heat gain or loss (W/m^2^).

$h_{c}$ is the convective heat transfer coefficient (W/m^2^·K).

$T_{surface}$ is the temperature of the internal wall surface (K).

$T_{air}$ is the temperature of the adjacent indoor air, not the external environment.

In our CFD simulations, $T_{air}$ and $T_{surface}$ are computed dynamically as part of the energy equation solution. The convective heat transfer coefficient $h_{c}$ is specified based on wall orientation and airflow conditions, typically ranging between 2 and 5 W/m^2^·K for indoor surfaces [41]. The wall is modeled as a solid conduction zone with internal heat generation (if any), and its surface temperature is coupled to the indoor airflow field. The calculated convective heat flux is automatically integrated into the energy balance by Airpak through boundary condition coupling and contributes to determining the final steady-state temperature field. According to Equation (5), if the wall temperature is higher than the ambient temperature, the heat loss from the room to the environment will be proportional to the temperature difference. Similarly, if the wall temperature is lower than the external temperature, heat will be transferred into the room. Conversely, if the heat flux at the wall is specified, the heat gain or heat loss of the wall will be independent of the temperatures of the wall and the environment. The heat transfer coefficient can be specified as a constant or as a function of temperature.

When setting the thermal balance conditions, the effects of air convection, thermal radiation, and heat sources (such as equipment and lighting) were considered. Taking into account the radiation heat sources from stage lighting and other equipment, the radiation heat transfer coefficient within the stage area was set, and the heat transfer was calculated based on the power of the heat sources (such as the power of the lighting fixtures). During the simulation process, the thermal load of the equipment on stage (such as lighting and audio equipment) was input as local heat sources. The heat generated by each piece of equipment was calculated using heat dissipation formulas. The specific thermal balance settings are shown in Table 3:

This study primarily considers the lighting heat source in the center of the stage performance area as the main heat source and only takes into account the thermal radiation in the negative *Y*-axis direction and along the *Z*-axis. All other settings use the default settings in Airpak.

#### 2.3.3. Meshing

In this study, we employed an adaptive mesh division strategy to ensure that the flow characteristics of the stage space are accurately captured. In the main areas of the stage space, particularly in the more regular rectangular areas (such as the main stage area), we used structured grids.

**Figure 2:**
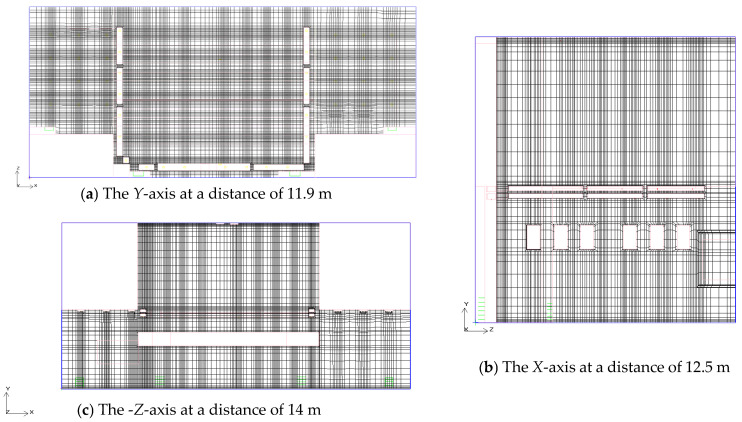
Stage space mesh quality. (In the picture, the orange color represents the exhaust duct, the blue color represents the air supply duct for the air conditioner, and the red color represents the return duct.).

The grid size within the stage space is relatively small, typically set between 0.05 m and 0.1 m. The mesh is further refined in areas such as the air supply outlets, exhaust outlets, and regions with concentrated airflow in order to capture local variations in airflow. Particularly in these areas, the grid size can reach 0.025 m to improve the local accuracy of the simulation. The grid quality is shown in Figure 2.

During the meshing process, we focused on refining the mesh in key areas while maintaining computational efficiency. Through the local mesh refinement strategy, the mesh density was increased in critical areas of the stage, particularly near the air supply outlets, exhaust outlets, and heat sources such as lighting, in order to more accurately simulate the airflow and thermal environment changes in these regions. Therefore, it can be concluded that the mesh in this study meets the computational requirements.

### 2.4. Evaluation Indices

In the study of airflow organization optimization, reasonable evaluation indicators are essential for accurately assessing the impact of different ventilation schemes on the stage’s thermal environment and airflow. In this study, several key evaluation indicators were set to comprehensively assess the optimized airflow patterns. These evaluation indicators include the uniformity coefficient, average temperature, and velocity vector diagrams. By analyzing these indicators collectively, improvements in thermal environment uniformity and airflow distribution resulting from the optimization measures can be visually demonstrated.

The uniformity index (UI) is a commonly used indicator to evaluate the uniformity of temperature or airflow distribution within the stage area. It reflects the degree of uniformity of the airflow and thermal environment. By calculating the uniformity index of temperature and wind speed in the stage area, the changes in the thermal environment and airflow distribution before and after optimization can be quantified. The uniformity index reflects the uniformity of the temperature and velocity fields, which means recording the temperature and velocity values at each measurement point in the indoor working area and calculating the average value of these values at *n* measurement points:

(3) $$\bar{t}=\frac{\sum t_{i}}{n},\;\bar{u}=\frac{\sum u_{i}}{n}$$

$\bar{t}$: average temperature, °C.

$t_{i}$: average temperature of the *i* measurement point, °C.

N: number of individual points measured.

Root mean square:

(4) $$\sigma_{t}=\sqrt{\frac{\sum ({t_{i}-\bar{t})}^{2}}{n}},\sigma_{n}=\sqrt{\frac{\sum {\left(u_{i}-\bar{u}\right)}^{2}}{n}}$$

$\bar{u}$: average speed, m/s.

$u_{i}$: the velocity of the *i* measurement point, m/s.

Unevenness coefficient:

(5) $$k_{t}=\frac{\sigma_{t}}{\bar{t}},k_{u}=\frac{\sigma_{u}}{u}$$

$\sigma_{t}$: temperature root mean square deviation.

$\sigma_{u}$: root mean square deviation of speed.

$k_{t}$: temperature inhomogeneity coefficient.

$k_{u}$: velocity inhomogeneity coefficient. These points are evenly arranged within the main occupied zone at a height of 1.5 m, which is widely accepted as the reference level for thermal comfort evaluation in large-volume spaces. The coefficient values are calculated from CFD post-processing data using Airpak’s “Point Sampling” tool and processed in Excel. A lower $k_{t}$ or $k_{u}$ indicates a more uniform distribution, and thus a more comfortable thermal environment.

Average temperature is one of the basic indicators of the thermal environment of a stage space, reflecting the overall temperature level within the stage area. In this study, the average temperature not only helps to evaluate the effect of airflow optimization on temperature but also helps to verify the effectiveness of ventilation strategies in reducing heat load and improving comfort. The calculation method is as follows:

(6) $$\bar{T}=\frac{1}{n}\sum_{i=1}^{n}T_{i}$$

$T_{i}$: temperature at the *i* th position in the stage area.

n: total number of measurement points. This indicator reflects the general trend of air temperature in the area of the stage.

Evaluation of ventilation effectiveness of fresh air systems in radiantly air-conditioned rooms was performed by using effluent efficiency [42]. Exhaust efficiency is quantified by calculating the ratio of the volume of air discharged by the exhaust system to the heat, pollutants, and volume of air produced in the stage space. For the purposes of this paper, the calculation is performed by treating heat as a pollutant as follows:

(7) $$\varepsilon =\frac{\left(y_{e}-y_{s}\right)}{\left(\bar{y}-y_{s}\right)}$$

$y_{e}$: concentration of pollutants at the exhaust vent.

$y_{s}$: concentration of pollutants in the fresh air stream.

$\bar{y}$: the average concentration of indoor pollutants. The higher the exhaust efficiency, the more effective the airflow system is at removing unwanted hot air and pollutants, helping to maintain fresh indoor air and temperature equalization.

A vector diagram of wind speed is a visualization tool to show the distribution and flow direction of airflow, which helps to intuitively understand the movement pattern of airflow under different ventilation schemes. Through the air velocity vector diagram, we can visualize the airflow direction, flow rate change, and potential airflow stagnation area in the stage area and further analyze whether the airflow distribution is reasonable and whether it can cover the performers and audience area. The wind speed vector map is generated by the airflow speed and direction in the simulation results. Each vector represents the wind speed and direction at one point. The air velocity vector diagram can help analyze the airflow direction and velocity distribution of the air supply and exhaust vents, as well as the existence of airflow dead spots and other problems.

## 3. Optimization Design Strategy

**Figure 3:**
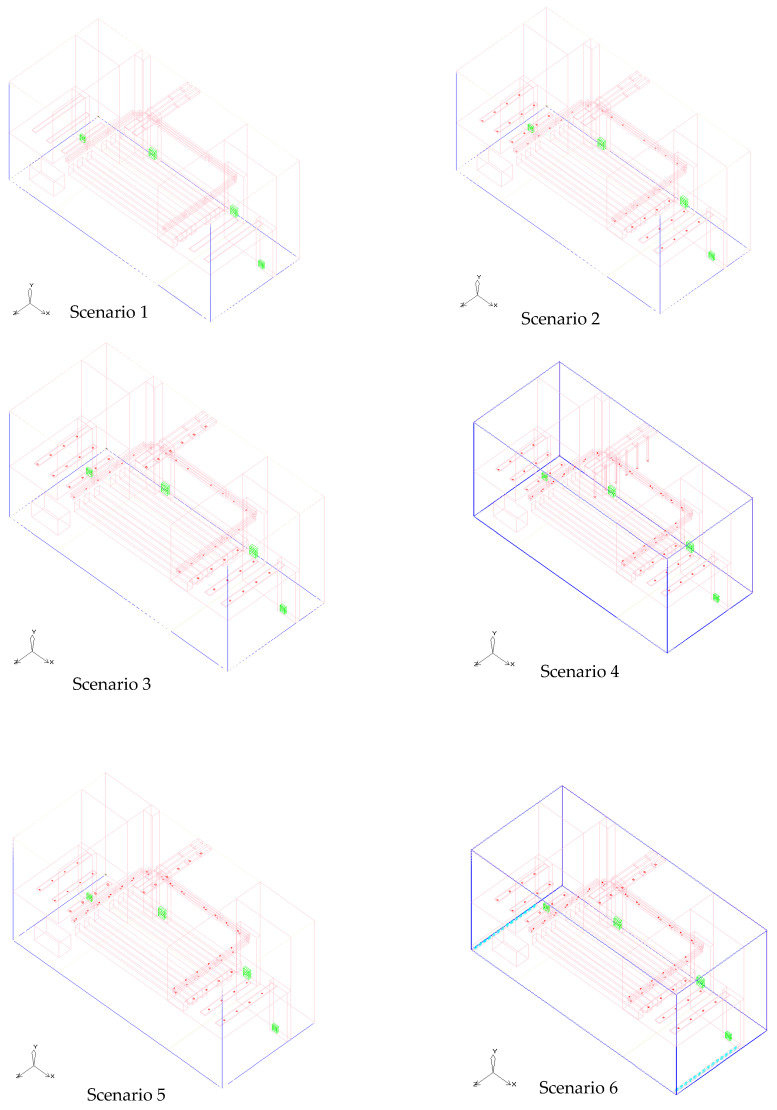
Working conditions and simulation scenarios. (In the figure, the blue color represents the calculation area, the deep red color represents the supply air outlets, the red color represents the blocks and pipes, the green color represents the exhaust air outlets, and the light blue color represents the fans.).

In order to comprehensively evaluate the optimization effect of different airflow organization strategies on the thermal environment of the stage, six typical working conditions were set up for comparative experiments in this study. These working conditions and scenarios were improved in terms of structure, equipment, and perturbation, respectively. The design features and variable parameters of each simulation scenario are shown in Table 4 and Figure 3.

Table 4 outlines the configuration details of the six simulation scenarios (S1–S6) designed to evaluate and improve airflow and thermal performance within the theater stage space. All scenarios were modeled under a constant total supply airflow condition to ensure comparability, while key parameters such as diffuser radius, distribution uniformity, and rear-stage airflow disturbance were selectively modified.

Scenario S1 serves as the baseline with uniform diffuser settings and no disturbance. Scenarios S2 to S4 introduce progressive refinements, including reduced or enlarged diffuser sizes and non-uniform layouts to redistribute airflow. Scenarios S5 and S6 further incorporate rear-stage airflow disturbances to simulate real-stage thermal and aerodynamic complexities. Scenario S6 represents the most optimized condition based on cumulative simulation feedback. This structured variation enables a comprehensive assessment of how geometric and distributional design factors affect indoor microclimate comfort.

## 4. Structural Improvement

The primary focus of structural improvements is on the ventilation structure layout of the stage space itself, with the objective of optimizing airflow distribution by modifying the natural ventilation effect of the stage. This type of improvement program encompasses the following two working conditions:

Scenario 1: In the event of the stage space being non-operational, all air supply outlets are to be closed, with airflow occurring exclusively via natural ventilation, i.e., without the involvement of a mechanical exhaust system. In such a scenario, the exhaust process is entirely reliant on external climatic factors, including wind speed, temperature difference, and other natural elements. The airflow distribution within the stage space is solely influenced by the external climate, thus eliminating the need for human intervention. The objective of this study is twofold: firstly, to evaluate the impact of natural ventilation systems devoid of mechanical exhaust support on airflow organization and the thermal environment; and secondly, to explore the limitations and applicability of natural ventilation in theater stage space.

Scenario 2: The absence of a mechanical exhaust system is counterbalanced by the integration of air supply and ventilation outlets. This configuration leads to enhanced airflow distribution through the strategic design of air supply and ventilation outlets. Notably, the optimization of airflow does not necessitate the implementation of a mechanical exhaust system. Instead, the design of reasonable air supply and exhaust paths serves to optimize the airflow within the system. The integration of air supply vents facilitates the introduction of fresh air into the stage area, while the ventilation vents serve to exhaust air, thereby achieving a specific thermal environment enhancement. This configuration enhances the rationality of the airflow path in comparison to Condition 1. However, it remains deficient in terms of the utilization of mechanical exhaust, and there is a possibility of excessive localized heat loads.

### 4.1. Equipment-Related Improvements

Equipment-based improvements focus on enhancing the effectiveness of airflow through the introduction of mechanical equipment, such as mechanical exhaust systems and air supply vents. The purpose of equipment-based improvements is to further improve the airflow distribution and temperature uniformity of the stage space by optimizing the layout and operation mode of the equipment. The following are the three types of working conditions:

Scenario 3: The case has been left in its original condition, complete with air supply, vents, and mechanical exhaust, all of which are in full working order. This case represents the original airflow organization scheme of the stage space, including the air supply, vent, and mechanical exhaust system. Through this basic scenario, the initial distribution of airflow can be observed, as well as the temperature and air velocity distribution in the stage space. This provides a benchmark for comparison for subsequent improvements.

Scenario 4: The functionality of the air supply and ventilation outlets has been verified, and the mechanical exhaust ducts have been extended to a length equivalent to half the height of the performance area within the stage space. In this instance, the mechanical exhaust system has been optimized through the extension of the length of the mechanical exhaust duct to half the height of the stage performance area. It is anticipated that this adjustment will facilitate enhanced overall airflow and mitigate the presence of elevated temperatures or heat retention in specific areas within the performance area of the stage. The objective of this enhancement is to optimize the functioning of the exhaust system and enhance the uniformity of the thermal environment.

Scenario 5: Keeping the total air supply volume unchanged, adjust the radius of the air supply outlet to regulate the temperature of the stage space. The specific operation is to increase the radius of the air supply outlet close to the stage performance area to 0.21 m, while reducing the radius of the air supply outlet in the waiting area to 0.12 m. The purpose of this case is to optimize the size and configuration of the air supply outlet, to improve the temperature distribution of the stage area, and to enhance the effect of local airflow control.

#### Disturbance-Type Improvement

Perturbation-type improvements focus on optimizing airflow and temperature distribution within a stage space through human physical intervention (e.g., adding a fan or changing the direction of airflow). This type of improvement does not rely on the addition or change of hardware, but rather on changing the local characteristics of the airflow to achieve optimization. The following are the perturbation-type improvement scenarios:

Scenario 6: Based on the original working conditions, two additional rows of fans were installed on each side of the waiting area of the stage space, keeping the position of the mechanical air vents unchanged. Each row of fans contains 14 fans with a radius of 0.4 m and an airflow of 0.75 m^3^/s. The artificial disturbance generated by these fans breaks the airflow stagnation and promotes the local airflow, thus improving the airflow uniformity and temperature distribution in the stage performance area. This work simulates the effect of physical intervention on the improvement of airflow distribution in the stage space and verifies the effectiveness of the fans in optimizing the stage thermal environment.

### 4.2. Research Subjects

The air-conditioning system of this project is designed with a variable refrigerant flow direct expansion air-conditioning system, in which combined air-conditioners are used in both the auditorium and the stage. The air-conditioning system of the main stage is equipped with inverter internal units, designed to supply air at 20 °C, and the equipment is placed in a special room, with the external units installed on the roof. The air supply mode of the main stage is side-feeding and down-feeding, the air supply outlets are equipped with air volume adjustment devices, and the return air outlets use single-layer louvered air outlets with filters. In order to avoid the influence of air-conditioning wind on the stage curtain, the indoor and outdoor units of the main stage are frequency conversion fans, the air supply pipeline is divided into upper and lower branches, the upper branch is used for side-feeding, and the lower branch is used for down-feeding, and the wind direction is adjusted through the electrically controlled double-position adjustment valve to ensure that air-conditioning effects are optimized in the course of the performance. The system in the air-conditioning season and the transition season uses the electronically controlled control valve to switch the air-conditioning mode. The air-conditioning season is calculated according to the per-person fresh air volume of 20 m^3^/h, and the transition season is used in the whole fresh air operation.

Considering that the stage area is as high as 27 m in height, the air conditioning system adopts a layered air supply method to guarantee the air conditioning effect in the lower area, and the top is equipped with a mechanical exhaust system for eliminating heat dissipation, and the volume of exhaust air is 80% of the volume of fresh air, and the inverter fan is linked with the air conditioning system to maintain the positive indoor pressure. The side stage adopts the airflow organization of upward sending and downward returning, and the air-sending and -returning outlets are round nozzles with adjusting devices and single-layer louvered air outlets.

The overall air-conditioning system enables precise control of the air-conditioning demand of each room through variable refrigerant flow regulation and achieves a balance between energy saving and a comfortable environment by adjusting the output of the mainframe through frequency conversion.

The maximum net height of the auditorium is 18 m. Considering the temperature stratification in the height direction, the air-conditioning system adopts the stratified air-conditioning system that guarantees the air-conditioning effect of the lower area, and the mechanical exhaust air is set up at the top to eliminate the heat dissipation of the upper part of the mechanical equipment and lights, etc., so as to realize energy saving. Top mechanical exhaust system mechanical exhaust volume of 80% of the fresh air volume, the use of variable frequency fan, fan, and air conditioning system chain control to ensure that the air conditioning conditions in the room maintain positive pressure.

**Figure 4:**
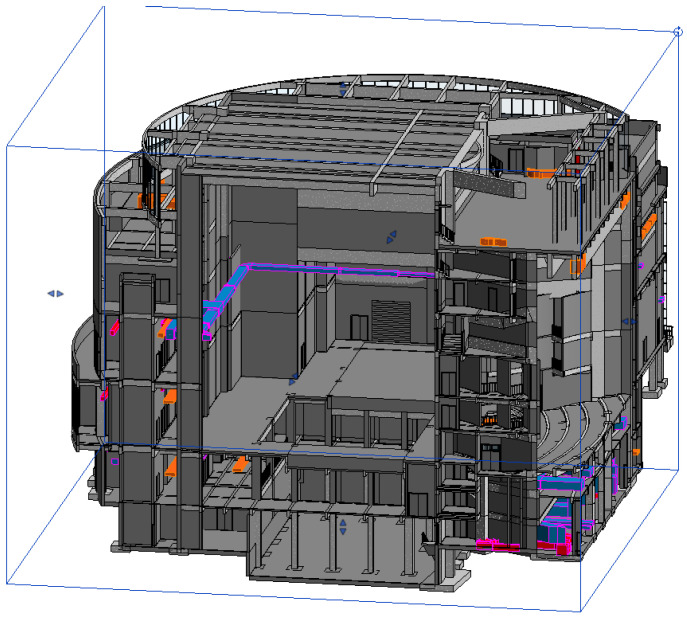
Stage effect diagram. (In the picture, the orange color represents the exhaust duct, the blue color represents the air supply duct for the air conditioner, and the red color represents the return duct).

**Figure 5:**
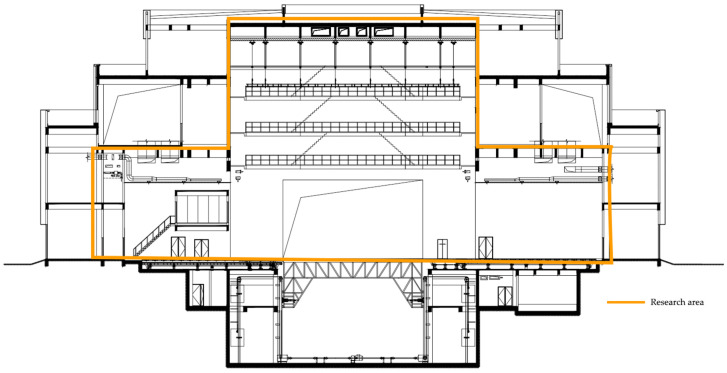
Stage cutaway.

The waiting area of the actors in Taicang adopts the air conditioning method of a heat recovery-type fresh air machine + indoor air duct machine, while the lounge, the backstage area of the actors, and the aisles adopt the air conditioning method of a unidirectional fresh air machine + indoor air duct machine. The airflow organization is upward-feeding and upward-returning, and the air supply outlet adopts double-layer louvered air with an air volume adjustment device, while the air return outlet adopts a single-layer louvered air outlet (with filter). Each indoor unit is equipped with a control panel, which can independently set and control various functions such as on and off control, operation condition setting, operation mode setting, temperature setting, air volume, and air direction switching. Variable refrigerant flow air-conditioning systems come with a more mature control method so that each room has less influence on each other. Through the temperature control device of each indoor terminal unit to regulate the operation of the unit, the outdoor host can be in accordance with the requirements of the indoor load changes, using frequency conversion to greatly adjust the host’s output, which can realize the precise control of the air-conditioning requirements of each room and achieve the maximum adjustment of the host’s output according to the requirements of the use of the host. It can realize precise control of the air conditioning requirements of each room and maximally adjust the output of the host according to the usage requirements, thus realizing the purpose of maximally adjusting the cooling capacity and saving energy. See Figure 4 and Figure 5.

## 5. Results

### 5.1. Temperature Distribution

**Figure 6:**
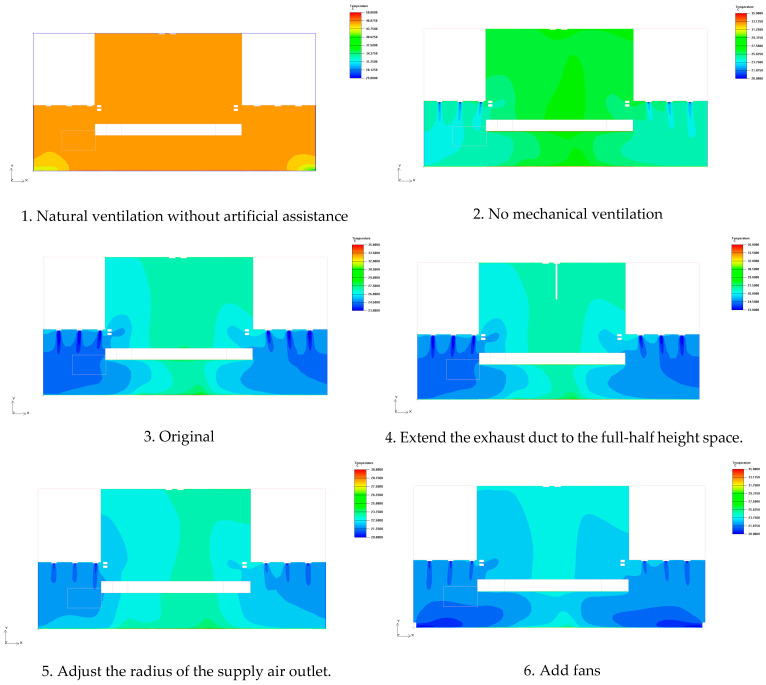
Front elevation at 17 m along the *Z*-axis.

**Figure 7:**
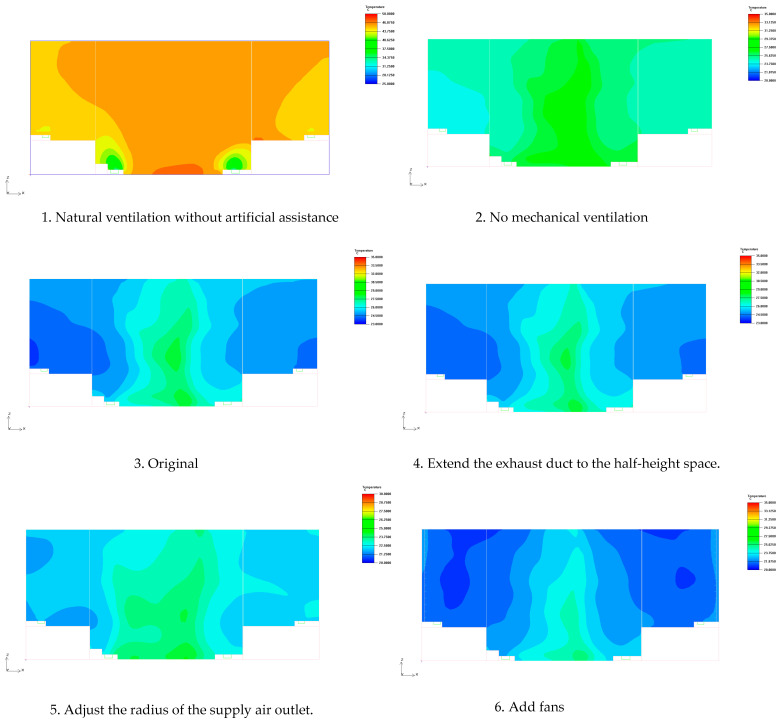
Front elevation at 1.5 m along the *Y*-axis.

Based on the comparative analysis of the temperature distributions of six typical working conditions in the front elevation at 17 m along the *Z*-axis and at 1.5 m along the *Y*-axis in the plane, the effects of different airflow organizations on the thermal environment of the stage space can be systematically evaluated. The experimental results show that the temperature fields of the stage space under different working conditions show significant differences. See Figure 6 and Figure 7.

The temperature distribution at 17 m along the *Z*-axis in the front facade for six typical working conditions is summarized for comparison, showing the influence of different airflow organization schemes on the temperature distribution of the stage space (Table 5).

This table clearly presents the different air flow organization methods under various working conditions and their impacts on the thermal environment of the stage. From the data in this table, it can be seen that the scheme of condition 6 (with added fan disturbance) is the best, significantly reducing the overall temperature and improving the uniformity of temperature distribution. While the optimization effects of the schemes relying solely on ventilation and mechanical exhaust (conditions 1–5) are all limited.

### 5.2. Comparison of Evaluation Index

**Figure 8:**
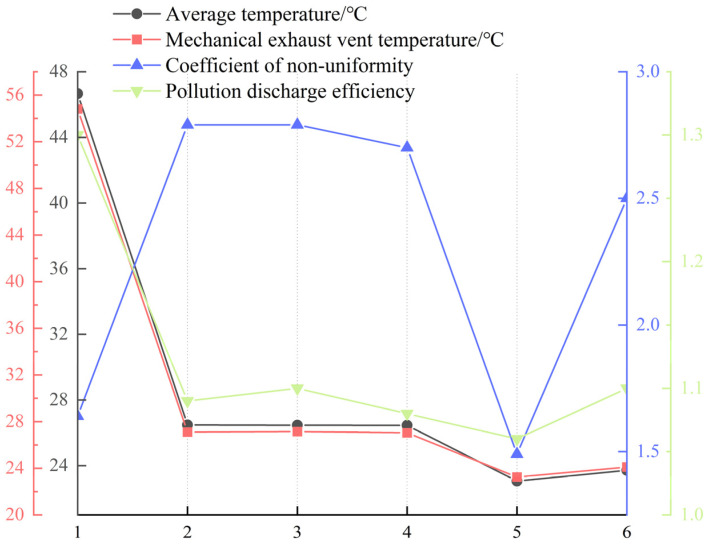
Evaluation indicators for six working conditions.

This study focuses on the uniformity of the thermal environment in the stage space of a theater, explores the influence of different airflow organization modes on the temperature distribution of the stage space, and analyzes the characteristics of the thermal environment under six typical working conditions through numerical simulation. This study shows that a single natural ventilation mode is difficult to effectively dissipate heat and even leads to heat accumulation and exhaust inversion phenomenon, while the traditional combination of air supply and mechanical exhaust still has limitations in improving the temperature distribution (Figure 8).

Compared to the original condition (Scenario 3), which represents the conventional mechanical ventilation approach, the proposed optimization strategies significantly enhance both temperature distribution and airflow performance. For instance, Scenario 5 and Scenario 6 reduced the overall stage temperature by approximately 3–5 °C and halved the temperature uniformity coefficient (from 2.79 to 1.49), while also increasing exhaust efficiency. These results quantitatively demonstrate that incorporating structural and disturbance-based adjustments offers notable improvements over standard ventilation configurations commonly used in stage design.

### 5.3. Vertical Distribution Analysis

**Figure 9:**
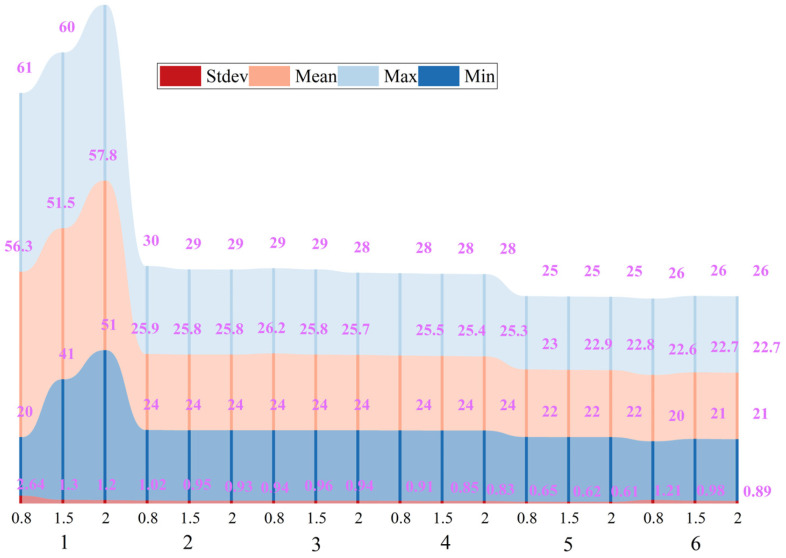
Influence of evaluation height on comfort analysis.

In this study, the primary simulation and evaluation were conducted at 1.5 m height, which corresponds to the thermal comfort-sensitive level for the human body, particularly in standing or seated positions [43]. This height is widely recognized in indoor environmental assessments as the most representative layer for occupant comfort. We further examined the temperature distribution at 0.8 m and 2.0 m height planes across all six scenarios, shown as Figure 9. The results demonstrate that while there are slight differences in mean temperature and standard deviation at these levels, the overall variation is minimal and does not significantly impact the interpretation of the airflow and thermal comfort performance. This consistency further supports the representativeness and reliability of using 1.5 m height as the evaluation baseline for thermal comfort analysis in large indoor spaces.

## 6. Discussion

### 6.1. Challenges and Obstacles

**Figure 10:**
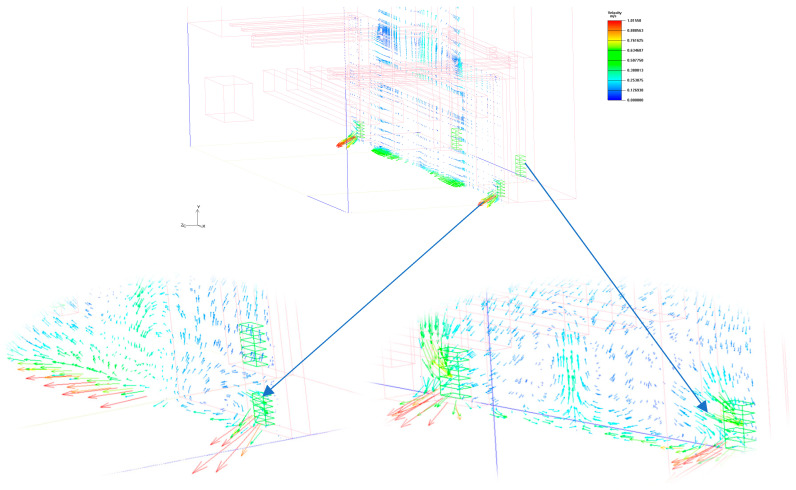
Limitations of natural ventilation. (The arrows in the picture represent the direction of wind speed movement, and the color ranges from blue to red to indicate the intensity of the wind speed.).

First, the limitations of natural ventilation are a major challenge. In Case 1, relying on natural ventilation alone does not effectively dissipate heat but instead leads to the phenomenon of backsiphonage at the air vents, creating a heat accumulation zone inside the stage space, as shown in the Figure 10. Since theater spaces usually have a tall building structure, the internal airflow is limited by thermal and wind pressure drivers, and it is difficult for natural ventilation to form a stable and uniform airflow organization in a complex indoor environment [44]. See Figure 10.

**Figure 11:**
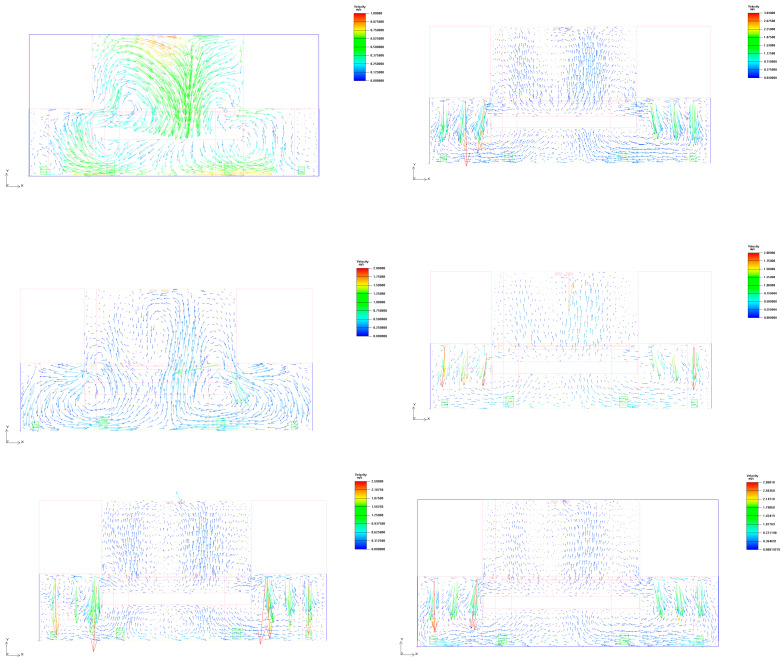
Wind speed vector. (The arrows in the picture represent the direction of wind speed movement, and the color ranges from blue to red to indicate the intensity of the wind speed.).

Secondly, the matching problem of air supply and exhaust air affects the temperature equalization of the stage area. From the experimental results of Case 2–4, the uniformity of the thermal environment is not significantly improved despite the increase in air supply and mechanical exhaust. The main reasons for this are as follows: 1. The height difference between the arrangement of air supply vents and exhaust vents leads to short-circuiting of the airflow, causing some of the high-temperature air to be trapped above the stage performance area. 2. The setting of the mechanical exhaust vents fails to effectively guide the hot air to dissipate, so the temperature distribution is still characterized by local inhomogeneity. 3. Although the mechanical exhaust ducts are extended to make the exhaust point closer to the high-temperature area in the stage space, the results of the experiments indicate that the mechanical exhaust ducts are still not sufficiently adjusted. This passive adjustment method still fails to adequately improve the thermal environment. See Figure 11.

In addition, the complexity of the theater space is also a key factor affecting the optimization of airflow organization. Theater stage spaces often have large spans, areas of varying heights, and complex equipment arrangements, and these structural characteristics increase the uncertainty of airflow [45]. At the same time, the stage performance process will generate a lot of heat (such as stage lighting, equipment cooling, etc.), further aggravating the unevenness of temperature distribution. Therefore, it is difficult to meet the comfort requirements of the stage area by only optimizing the airflow organization through traditional mechanical exhaust means.

### 6.2. The Role of Mechanical Disturbance

In addressing the challenges and obstacles mentioned above, a key role in optimizing the airflow organization and improving the uniformity of the thermal environment was played by adjusting the radius of the air supply opening and mechanical disturbance (mechanical disturbance). In Condition 5, the temperature distribution in the stage space was significantly improved. After increasing the radius of the air supply outlet in the performance area to 0.21 m and decreasing the radius of the air supply outlet in the waiting area to 0.12 m, the temperature in the stage performance area is significantly reduced and the airflow distribution is more uniform. From the experimental results of working condition 6, on the basis of the original working condition, after adding fans on both sides of the waiting area, the temperature field of the stage space is significantly improved, as shown in Figure 1. Specific performance is as follows: 1. Overall temperature reduction: experimental data show that the average temperature of the stage space went from 26 °C to 21.0 °C to achieve an effective cooling effect. 2. Temperature distribution is more uniform; the average temperature is reduced to 23 °C, indicating that the high-temperature region is significantly reduced, and the temperatures of stage performance and waiting areas tend to be the same. 3. Eliminating the local heat buildup: the introduction of the fan to promote the mixing of the airflow to make the high-temperature air masses effectively diffused so as to avoid the heat accumulation. 4. The fan can be used in the stage space. The introduction of fans promoted airflow mixing so that high-temperature air masses could be effectively diffused, avoiding the phenomenon of high temperature in the performance area and low temperature on both sides.

The advantage of mechanical perturbation is that it actively controls the airflow and enhances the mixing of the air so that it is more evenly distributed across the stage space. This type of optimization is more adaptable than relying solely on mechanical exhaust or supply air and can be flexibly adjusted to the actual demands of the thermal environment inside the theater [46]. From the engineering application point of view, in the airflow optimization design of the theater stage space, the appropriate addition of mechanical disturbance devices (such as fans or infusion devices) can effectively improve the airflow pattern and enhance thermal comfort, thus creating a better thermal environment for the performers and the audience.

### 6.3. Limitations

Although the proposed multi-scenario airflow optimization strategy based on CFD simulation has yielded promising results, several limitations must be acknowledged:

(1) Steady-state simulation assumption: The CFD model used in this study is based on steady-state conditions, which do not account for time-varying thermal loads or dynamic factors such as fluctuating occupancy, performance duration, or equipment usage cycles. These elements may influence real-world airflow patterns and thermal performance.

(2) Simplified boundary conditions: To ensure simulation feasibility, some boundary parameters—such as external wind pressure, solar radiation, and infiltration through openings—were simplified or assumed constant. This may introduce deviations when applied to real-world environments with fluctuating boundary conditions.

(3) Case-specific applicability: The optimization scheme was designed based on a specific theater stage layout, mechanical system configuration, and geometric constraints. As such, its direct applicability to other types of large spaces (e.g., exhibition halls, gymnasiums) may be limited without further adaptation.

These limitations provide avenues for further improvement, such as dynamic transient simulations, incorporation of occupant behavior modeling, and cross-validation using experimental or sensor-based field data.

## 7. Conclusions

1. In tall, enclosed spaces (such as theaters, exhibition halls, etc.), the efficiency of natural ventilation is usually limited by external climatic conditions and the layout of the building structure. Especially in the case of higher temperatures or lower wind speeds, a single natural ventilation is often difficult to meet the demand for a balanced airflow and thermal environment in the stage space. In this context, this study innovatively combines a hybrid system of mechanical and natural ventilation and optimizes the airflow distribution effect by adjusting the radius of the air supply outlet and the exhaust path.

2. This study proposes to proactively improve the indoor thermal environment and airflow by means of internal airflow regulation (e.g., physical interventions such as radius adjustment of air supply outlets, extension of mechanical exhaust ducts, addition of fans, etc.). The introduction of this internal drive mechanism provides a new way of thinking to improve space thermal comfort and air quality.

3. Tall, enclosed spaces such as theaters and exhibition halls usually have problems of heat load accumulation and airflow stagnation due to the height of the space and the characteristics of confinement. Traditional ventilation design is difficult to meet the airflow distribution and temperature control needs of such large spaces. By introducing airflow organization optimization strategies into the design, such as adjusting the location of air supply outlets, enlarging the radius of air supply outlets, and setting up multi-point ventilation paths, this study is able to achieve a more uniform airflow distribution and thermal environment optimization in large spaces.

4. This study proposes a comprehensive optimization scheme for the stage space through numerical simulation and actual case analysis, combining optimization means in the three aspects of structure, equipment, and perturbation. Particularly, in terms of perturbation, by setting up physical interventions such as fans, this study investigates how to break airflow stagnation and accelerate airflow by artificially perturbing the airflow so as to improve the local temperature distribution and airflow uniformity. This innovative perturbation provides a more flexible and efficient means of airflow optimization based on traditional equipment regulation and structural optimization schemes. The effect of mechanical disturbance on temperature uniformity improvement is remarkable: on the basis of the original working conditions, after adding fans on both sides of the waiting area, the average temperature of the stage space dropped from 27 °C to 24 °C, with a more uniform temperature distribution, indicating that the disturbance effect produced by the fans can promote air mixing and improve the uniformity of the thermal environment. It provides a new possibility for the internal drive of ventilation in large public buildings in the future.

## Figures and Tables

**Table 1:** Air supply velocity.

Name	Velocity/(m/s)	Direction
Opening.1-1 to opening.3-4	−5.18	Y
Opening.4-1 to opening.4-6	4.15	X
Opening.5-1 to opening.5-6	−4.51	Y
Opening.6-1 to opening.6-8	4.51	Z
Opening.7-1 to opening.7-8	−4.51	Y
Opening.8-1 to opening.8-6	−4.51	X
Opening.9-1 to opening.9-6	−4.51	Y
Opening.10-1 to opening.12-4	−5.18	Y
Opening.13-1 to opening.13-6	3.2	Y

**Table 2:** Thermal boundary conditions within the calculation domain.

Name	Wall Type	Outside Heat Flux/(W/m^2^)
Left floor	Stationary	40.0
Mid-floor	Stationary	110.0
Right floor	Stationary	40.0
Room-side Y max	Stationary	9.88

**Table 3:** Thermal boundary conditions within the calculation domain from stage lighting and other equipment.

Min. Y Fixed Heat (W/m^2^)	Min. Z Fixed Heat (W/m^2^)	Max. Z Fixed Heat (W/m^2^)
156.0	10.0	10.0

**Table 4:** The design features and variable parameters of each simulation scenario.

Scenario	Supply Air Volume	Diffuser Radius	Rear-Stage Airflow Disturbance	Configuration Notes
S1	Constant	Uniform (R = 0.15)	None	Baseline condition
S2	Constant	Reduced	None	Reduced airflow configuration
S3	Constant	Increased	None	Enlarged outlet radius
S4	Constant	Non-uniform	None	Variable radius by zone
S5	Constant	Non-uniform	With disturbance	Disturbance air added
S6	Constant	Optimized	With disturbance	Final improved design

**Table 5:** The temperature distribution along the *Y*-axis at the 17-meter position on the main facade.

Number	Organization	Scale Range/(°C)	Feature	$k_{t}$
1	Only natural ventilation	25.0–50.0	The exhaust vent is sucking in air, causing the heat to accumulate and the temperature to soar extremely high.	0.030
2	No mechanical ventilation	20.0–30.0	Low-efficiency heat dissipation—the temperature has decreased somewhat but is still uneven.	0.037
3	Original	23.0–35.0	The temperature in the waiting area is low (24 °C), while that in the performance area is high (27 °C), resulting in uneven temperature distribution.	0.036
4	Extend to the full-half height space	23.0–35.0	The heat dissipation has slightly increased, but the temperature distribution remains uneven.	0.033
5	Adjust the radius of the supply air outlet	20.0–30.0	The temperature is evenly distributed, and it can effectively improve the thermal environment of the stage space.	0.027
6	Add fans	20.0–35.0	The overall temperature dropped, the temperature distribution became uniform, and the thermal environment of the stage was significantly improved.	0.045

## Data Availability

Data are contained within the article.

## Abbreviations

The following abbreviations are used in this manuscript:

ADPI	Air Diffusion Performance Index
AIQ	indoor air quality
B-G Model	Block–Gebhart Model
CBAD	Ceiling-Based Air Distribution
CFD	computational fluid dynamics
HVAC	Heating, Ventilation, and Air Conditioning
IAQ	indoor air quality
PMV	Predicted Mean Vote
RCI	Rack Cooling Index
RH	Relative Humidity
RTI	Return Temperature Index
UI	uniformity index
UFAD	underfloor air distribution
CFD-R	CFD Radiation Model
ε-κ Model	Turbulence Model (Turbulent Dissipation Rate–Kinetic Energy Model)

## Appendix A. Governing Equations Used in CFD Simulation

For reference and reproducibility, the main governing equations used in the CFD simulations conducted with Airpak software are provided here.

The expression of the continuity equation is as follows:

(A1) $$\frac{\partial \rho }{\partial t}+\nabla \cdot \left(\rho \nu \right)=0$$

ρ: density of fluid.

ν: fluid velocity.

The energy equation for the fluid region can be expressed as enthalpy of sensible heat. $h(h=\int_{{Τ}_{ref}}^{\tau }c_{\rho }dT$, $T_{ref}$ is 298.15 K) is expressed in the following form:

(A2) $$\frac{\partial }{\partial t}\left(ph\right)+\nabla \cdot \left(ph\nu \right)=\nabla \cdot \left[\left(k+k_{t}\right)\nabla T\right]+S_{h}$$

ρ: density, kg/m^3^.

h: the total energy possessed by a unit mass of fluid, J/kg.

k: the thermal conductivity of molecules, W/(m·K).

$k_{t}$: due to the thermal conductivity resulting from turbulent transport ($k_{t}=c_{\rho }\mu_{t}Pr_{t}$).

$S_{h}$: includes all the user-defined volume heat source terms.

T: temperature, K.

In the solid conduction region, the heat conduction equation solved by Airpak is shown in Equation (1), which includes the heat flux density generated by both internal conduction and volumetric heat sources:

(A3) $$\frac{\partial }{\partial t}\left(ph\right)=\nabla \cdot \left[k\nabla T\right]+S_{h}$$

By simultaneously solving Equation (1) with the energy transport Equation (A5) for the flow region, the predicted distribution of conduction/convection heat transfer under the fluid–structure coupling can be obtained.

In the standard k−ε model, the turbulence kinetic energy (*k*) and the turbulence dissipation rate (ε) can be obtained from the following transport equations:

(A4) $$\frac{\partial }{\partial t}\left(\rho k\right)+\frac{\partial }{{\partial x}_{i}}\left(\rho {ku}_{i}\right)=\frac{\partial }{{\partial x}_{i}}\left[\left(\mu +\frac{\mu_{i}}{\sigma_{k}}\right)\frac{\partial k}{{\partial x}_{i}}\right]+G_{K}+G_{b}-\rho \varepsilon$$

(A5) $$\frac{\partial }{\partial t}\left(\rho \varepsilon \right)+\frac{\partial }{{\partial x}_{i}}\left({\rho \varepsilon u}_{i}\right)=\frac{\partial }{{\partial x}_{i}}\left[\left(\mu +\frac{\mu_{t}}{\sigma_{\varepsilon }}\right)\frac{\partial \varepsilon }{{\partial x}_{i}}\right]+C_{1\varepsilon }\frac{\varepsilon }{k}\left(G_{k}+C_{3\varepsilon }C_{b}\right)-C_{2\varepsilon }\rho \frac{\varepsilon^{2}}{k}$$

$G_{k}$: the generation term of turbulence kinetic energy (k) caused by the mean velocity gradient.

$C_{b}$: the generation term of turbulence kinetic energy (k) caused by buoyancy.

$C_{1\varepsilon }$, $C_{2\varepsilon }$, and $C_{3\varepsilon }$ are constants.

$\sigma_{k}$ and $\sigma_{\varepsilon }$ are the turbulence Prandtl numbers for k and ε, respectively.

These equations describe the airflow process within the theater stage space and incorporate the effects of heat sources (such as lighting, stage equipment, and the thermal load from performers).
